# Utilization of Imaging in the Diagnosis and Surveillance of Pancreatic Ductal Adenocarcinoma (PDAC): Analysis of the TRICARE Data

**DOI:** 10.7759/cureus.110968

**Published:** 2026-06-16

**Authors:** Yun Jee Kang, Mahima Jain, George Molina

**Affiliations:** 1 Center for Surgery and Public Health, Department of Surgery, Brigham and Women’s Hospital, Boston, USA; 2 Center for Aging and Serious Illness, Department of Palliative Care, Massachusetts General Hospital, Boston, USA

**Keywords:** abdominal mri, abdominopelvic ct scan, pdac (pancreatic ductal adenocarcinoma), postoperative surveillance imaging, pre-treatment imaging

## Abstract

Background

It is unclear if adding MRI in the pre-treatment staging for pancreatic ductal adenocarcinoma (PDAC) is associated with changes in treatment compared to only obtaining a CT scan of the abdomen/pelvis. Similarly, there remains unclear benefit from frequent postoperative surveillance imaging after surgical resection for PDAC.

Methodology

Claims data from the Military Health Services Data Repository were used to identify all adult patients with PDAC who underwent surgical resection between 2017 and 2022. Patient demographics, medical and surgical interventions, radiographic studies, and survival data were sourced from the TRICARE database. The proportion of patients undergoing neoadjuvant chemotherapy compared to upfront surgical resection was compared between patients with and without a preoperative abdominal MRI, along with those who underwent one versus multiple preoperative CTs, after controlling for covariates of interest. Overall survival was compared between patients who underwent surveillance imaging every six months versus those with more frequent imaging.

Results

There was no significant difference in whether patients who had preoperative MRIs underwent surgery first (88.5% vs. 77.9%, p = 0.58). However, among patients who received multiple CT scans, there was a higher proportion who underwent surgical resection before chemotherapy (92.2% vs. 74.3%, p = 0.017). Decreased surveillance intervals were associated with a decreased hazard ratio (0.88, p = 0.007) when compared to the six-month standard surveillance frequency, although only after the 24-month mark.

Conclusions

Overall, preoperative MRIs were not associated with initial choice of treatment (i.e., surgery first or chemotherapy first). Frequency of surveillance imaging might differ before and after the 24-month mark, but disentangling the reason for imaging in the post-surgical surveillance period is difficult and introduces bias.

## Introduction

Overutilization of imaging remains a significant contributor to wasteful healthcare spending despite numerous guidelines and recommendations aimed at reducing low-value care [1-3]. Studies have found that as many as 20-50% of high-technology imaging procedures (computed tomography (CT), magnetic resonance imaging (MRI), positron emission tomography (PET), etc.) fail to offer clinically significant information [4-6]. Many patients undergo multiple diagnostic imaging examinations for the same disease process, despite these additional examinations providing limited to no impact on treatment decisions.

Clinical practice guidelines for pancreatic ductal adenocarcinoma (PDAC) recommend a pancreatic protocol CT (multi-phase, contrast-enhanced, thin slice) as the preferred imaging modality for diagnosis [7,8]. Patients occasionally also receive an MRI or other imaging studies as part of their initial workup, as several studies have shown that MRIs can be more sensitive than CT for the identification of liver metastases; however, it is unclear whether these supplementary examinations truly result in changes in clinical management. Regarding postoperative surveillance, National Comprehensive Cancer Network (NCCN) guidelines previously recommended CT scans every three to six months for the first two years following resection; however, data surrounding this has been scarce, and there has been no clear consensus on surveillance imaging internationally. The most recently updated NCCN guidelines continue to recommend surveillance imaging but do not recommend at which rate this should occur [7,9]. Retrospective data have shown that regular surveillance including both clinical and radiographic assessment identifies patients with asymptomatic recurrence, and early detection may offer a therapeutic window in which patients may receive additional treatment [10-12]. On the other hand, no proven curative treatments for recurrent PDAC exist, and there is limited evidence that early identification of recurrence or metastasis has an impact on outcomes [8,13,14]. Therefore, the utility of undergoing CT scans as frequently as every three months in the absence of symptoms or an increasing cancer antigen 19-9 (CA 19-9) level is unknown.

We used claims data from the Military Health System (MHS), which provides universal insurance coverage to active-duty military service members, military retirees, reserve members, and eligible dependents, to explore whether receiving these ancillary imaging tests was correlated with improved patient outcomes or was a potential source of low-value care for patients with PDAC. We aimed to examine whether patients who underwent a secondary radiologic examination, such as MRI, had different rates of upfront surgical resection compared to those who only received a CT, and whether more frequent post-treatment surveillance of patients with a recent history of PDAC was associated with improved survival.

## Materials and methods

This study utilized claims data from the MHS Data Repository between 2017 and 2022. We used the following datasets within the TRICARE database: direct care inpatient, private care inpatient, direct care outpatient, and private care outpatient. Radiology examination data and demographic information were also obtained.

We identified patients who were 18 years or older with clinically staged T1 through T3 pancreatic adenocarcinomas using International Classification of Diseases, 10th Revision, Clinical Modification (ICD-10-CM) diagnosis codes (Appendices). T4 tumors were excluded because they are considered locally advanced (i.e., according to the American Joint Committee on Cancer 8th edition, these are defined as having major arterial involvement). We excluded these because they are often treated with neoadjuvant chemotherapy as the initial treatment. We further isolated those who underwent surgical resection using ICD-10 PCS codes (Appendices). Patients with missing information about diagnosis, T staging, surgery, or chemotherapy were excluded. Patients with no information about imaging were excluded.

Upfront treatment was defined as the first listed treatment (surgery or chemotherapy) from the time of diagnosis to up to four months later. Among this cohort, we identified patients who underwent pre-treatment CT alone versus CT plus MRI. All patients needed to have received at least one of these imaging modalities. Imaging before surgery was defined as CT of the abdomen/pelvis and/or MRI of the abdomen up to four months before surgery. Imaging before neoadjuvant therapy was defined as CT of the abdomen/pelvis and/or MRI of the abdomen up to four months before initiation of chemotherapy. Chi-square test was used, and Fisher’s exact test was used if any cell was less than 10, to compare the difference in receiving neoadjuvant chemotherapy or upfront surgical resection among patients who received at least one CT scan versus more than one CT scan and among patients who received an MRI versus patients who did not receive an MRI. We also compared the difference in receiving a CT scan plus an MRI versus only a CT scan before initial treatment between women and men.

A multivariable logistic regression adjusting for age group, sex, and race was fitted to evaluate the relationship between receiving upfront surgical resection versus upfront chemotherapy among patients who received one versus more than one pre-treatment abdominopelvic CT scan.

Post-treatment surveillance

All patients who underwent surgery were included, and we identified all post-surgery imaging that they received. For the surveillance imaging, we captured all imaging that was done after surgery was performed. We then calculated the time interval from surgery and the previous imaging study (if one was done) to identify the imaging interval. For example, if surgery was performed in March 2022 and the first imaging was in June 2022, then the imaging interval for that first imaging study was three months. If the second imaging was then performed in October 2022, then the imaging interval for the second imaging study was four months (October 2022-June 2022). We did this for all patients who had surgery and had post-surgery surveillance imaging. We excluded all imaging in the surveillance imaging analysis that was done before surgery. Among the entire cohort and according to racial groups, we calculated the median interval in months of the frequency of the surveillance imaging. The Kruskal-Wallis test was used to compare differences in median intervals in months.

Overall survival (OS) was analyzed using multivariable Cox proportional hazards regression models that were adjusted for age group, race, and sex, and with time-varying treatment effects. To account for non-proportional hazards, the treatment effect was modeled using a piecewise approach with a prespecified change point at 24 months following diagnosis. Follow-up time was divided into two intervals (≤24 months and >24 months), and separate treatment hazard ratios (HRs) were estimated for each interval using start-stop counting process notation. HRs and 95% confidence intervals (CIs) were estimated separately for early (≤24 months) and late (>24 months) follow-up intervals.

To estimate adjusted median OS, marginal standardized curves were generated from the fitted Cox model. Briefly, predicted survival probabilities were calculated for each individual under the following two hypothetical treatment scenarios: (1) all individuals had surveillance imaging that was less frequent than every six months, and (2) all individuals had surveillance imaging that was more frequent than every six months, while retaining each individual’s observed covariate profile. Predicted survival curves were then averaged across the study population to generate covariate-adjusted marginal survival curves for each surveillance imaging frequency group. Adjusted median OS was defined as the time at which the marginal standardized survival probability crossed 0.50.

A 24-month cutoff was selected because at our institution we routinely obtain surveillance imaging every three months during the first two years, and then after the two-year mark, we extend the surveillance imaging interval to six months. According to the previous NCCN guidelines, the recommendation was to obtain surveillance imaging three to six months for the first two years and then extend to six months after two years. Time of follow-up was defined as the time from the date of surgery to death from any cause or the last date of follow-up. All analyses were performed using SAS version 9.4 (SAS Institute Inc., Cary, NC, USA).

## Results

We identified 184 patients with pancreatic adenocarcinomas and without evidence of distant metastasis who were diagnosed from 2017 to 2022 and underwent surgical resection. The preoperative imaging cohort included 138 patients, and the postoperative surveillance imaging cohort included 125 patients (Appendices). Table 1 summarizes these patient characteristics. Of these patients, 54.4% were male and 45.7% female. White patients comprised 46.7% of the cohort, while black patients represented 20.1%, and Asian, other, and unknown made up the remaining 33.2%.

**Table 1:** Patient characteristics. The data has been represented as N (%).

	Entire cohort, N = 184	Preoperative imaging cohort, N = 138	Postoperative surveillance imaging cohort, N = 125
Age group (years)			
18–34	12 (6.5%)	8 (5.8%)	10 (8.0%)
35–44	16 (8.7%)	9 (6.5%)	11 (8.8%)
45–64	95 (51.6%)	75 (54.3%)	59 (47.2%)
≥65	61 (33.2%)	46 (33.3%)	45 (36.0%)
Sex			
Female	84 (45.7%)	62 (44.9%)	59 (47.2%)
Male	100 (54.3%)	76 (55.1%)	66 (52.8%)
Race			
Asian, other, and unknown	61 (33.2%)	43 (31.2%)	38 (30.4%)
Black	37 (20.1%)	28 (20.3%)	25 (20.0%)
White	86 (46.7%)	67 (48.6%)	62 (49.6%)

Of the 184 patients, 138 had preoperative imaging information. The majority of these patients underwent at least one preoperative abdominopelvic CT scan (53.6%), while 37% underwent more than one abdominopelvic CT scan (Table 2). Overall, 44.2% of patients had an abdominal MRI. All patients who did not have an abdominal MRI had undergone at least one abdominopelvic CT scan. There were 13 patients who did not receive a CT scan, and all these patients received an abdominal MRI.

**Table 2:** Imaging modality before first treatment (surgery versus chemotherapy). The data have been represented as N (%).

Abdominal/Pelvic CT scan	N = 138
Received one CT scan	74 (53.6%)
Received more than one CT scan	51 (37.0%)
Did not receive a CT scan	13 (9.4%)
Abdominal MRI	
Received an MRI scan	61 (44.2%)
Did not receive an MRI scan	77 (55.8%)

No significant difference was found between men and women in the proportion of patients who received an abdominopelvic CT scan as well as an MRI versus only an abdominopelvic CT scan before their initial treatment (p = 0.58, chi-square test). Patients who received more than one pre-treatment abdominopelvic CT scan were more likely to undergo surgery as their initial treatment rather than chemotherapy (92.2% vs. 7.8%) when compared to those who received only one abdominopelvic CT scan (74.3% vs. 25.7%) (p = 0.02, Fisher’s exact test) (Table 3). There was no difference in the proportion of patients who underwent upfront surgical resection or upfront chemotherapy among patients who received an MRI compared to those who did not (p = 0.12, Fisher’s exact test). After adjusting for age groups, sex, and race, patients who received more than one pre-treatment abdominopelvic CT scan had higher odds of undergoing surgical resection (adjusted odds ratio (OR) 4.6, 95% CI = 1.3-15.6, p = 0.01) (Table 4).

**Table 3:** Association between imaging modality and first treatment (surgery versus chemotherapy). The data have been represented as N (%). A p-value <0.05 was considered statistically significant. *: Fisher’s exact test was used in the univariable analysis due to at least one cell size that was less than 10.

	Upfront surgical resection	Upfront chemotherapy	P-value*
Received one CT scan	55 (74.3%)	19 (25.7%)	0.02
Received more than one CT scan	47 (92.2%)	4 (7.8%)	
Received an MRI	54 (88.5%)	7 (11.5%)	0.12
Did not receive an MRI	60 (77.9%)	17 (22.1%)	

**Table 4:** Multivariable logistic regression model evaluating for association between receiving one versus more than one abdominopelvic CT scan and odds of undergoing surgical resection. *: Multivariable logistic regression was adjusted for age group, sex, and race. Age groups were collapsed due to low sample size (age groups collapsed to 18-44, 45-64, and 65 and greater). A p-value <0.05 was considered statistically significant. OR = odds ratio; CI = confidence interval

	Adjusted OR	95% CI	P-value*
Received one CT scan (reference)			
Received more than one CT scan	4.6	1.3-15.6	0.01

We then examined surveillance trends after surgery in this population. Overall, 125 out of 184 had data to test our hypothesis. The median interval in surveillance imaging after surgical resection was three months for the entire cohort. White patients had lower median intervals (two months), compared to black patients (three months), and Asian/other patients (three months) (p = 0.05) (Table 5).

**Table 5:** Interval in surveillance imaging after surgical resection for pancreatic cancer. A p-value <0.05 was considered statistically significant. The Kruskal-Wallis test was used to compare differences in median intervals in months *: P-value = 0.05.

	Median (interquartile range (25^th^–75^th^))
Entire cohort	3 months (1–5 months)
By race*	
Black	3 months (1–4 months)
White	2 months (1–4 months)
Asian, other, and unknown	3 months (1–5 months)

In multivariable Cox regression analyses incorporating time-varying treatment effects, the association between treatment and mortality differed between the early (≤24 months) and late (> 24 months) follow-up periods. During the first 24 months following surgery, receiving a surveillance scan every <6 months was associated with a higher hazard of death compared with receiving a surveillance scan every ≥6 months (adjusted HR = 4.9, 95% CI = 1.7-13.7). The effect of the frequency of receiving a surveillance scan beyond 24 months could not be estimated because of insufficient events among the patients at risk after 24 months.

**Figure 1:**
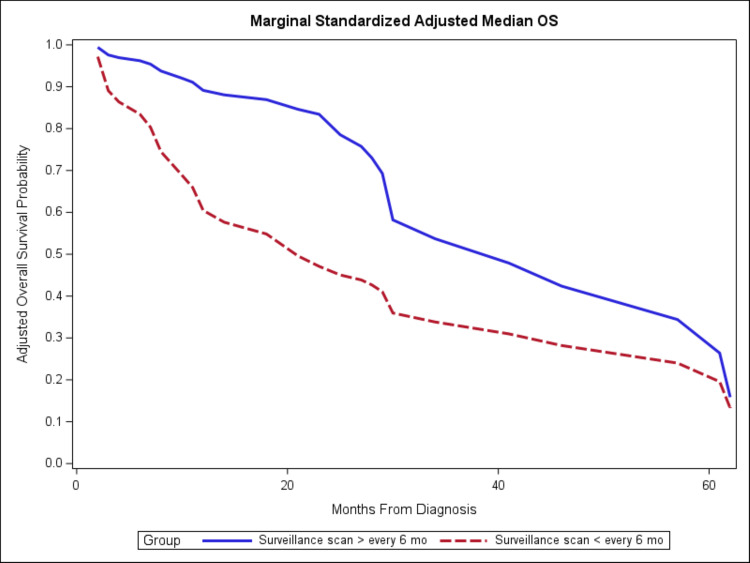
Adjusted survival curves for the two surveillance groups. Solid line group: Patients who received surveillance scans more frequently than every six months. Dashed line group: Patients who received surveillance scans less frequently than every six months. Predicted survival curves were averaged across the study population to generate covariate-adjusted marginal survival curves. OS = overall survival

Marginal standard survival analyses demonstrated an adjusted median OS of 21 months for patients who received a surveillance scan every <6 months compared with 41 months for patients who received a surveillance scan every ≥6 months. Adjusted survival curves for the two surveillance groups diverged during the first 24 months following surgery and subsequently demonstrated attenuation thereafter (Figure 1).

## Discussion

We found that a significant number of patients undergo additional pancreatic cancer imaging with MRI, but that this was not associated with a change in initial treatment choice (i.e., upfront surgical resection versus upfront chemotherapy). This might suggest that MRIs are unlikely to affect clinical decision-making, and use of this imaging modality should be more carefully considered with the goal of reducing unnecessary testing and cost to patients and the healthcare system. However, it is important to highlight that our findings might reflect selection bias rather than true clinical impact. We did identify an association between an increased number of pre-treatment abdominopelvic CT scans and patients who underwent upfront surgical resection. This merits further exploration of the factors that might be driving this association, such as use of a pancreas protocol CT to delineate resectability following an initial finding on a standard abdominopelvic CT scan, which might be driven by surgeon and/or institution preferences.

In addition, we found lower OS among patients who received more frequent surveillance imaging. The etiology of this association is unclear, although this could represent patients who received imaging earlier than planned due to symptoms from early recurrence/metastasis. Although the adjusted survival curves diverged during the first 24 months following surgery, they subsequently demonstrated attenuation after the 24-month mark. This might reflect detection of asymptomatic recurrence after the 24-month mark that may benefit from additional aggressive treatment or recruitment into novel clinical trials, as suggested by other prior studies [10,12,15]. However, our findings might be partially biased by residual confounding, heterogeneous follow-up, selection bias, reverse causation, unmeasured confounding that is inherent to claims-based analyses, and missing data.

The use of pre-treatment imaging is important due to potential overuse of imaging that can lead to harm and can add to costs of care. For example, more than a third of patients being worked up for an adrenal mass underwent a secondary imaging modality, even though more than 90% of these patients had already met the criteria for an adrenalectomy after the initial imaging procedure and biochemical screening [16]. In addition to increased costs to patients and healthcare systems, this overuse can also lead to potential harm by way of unnecessary radiation exposure, false-positive or negative test results, and incidental findings. Neither the NCCN nor the American Congress of Obstetricians and Gynecologists recommends imaging for patients with low-grade uterine cancer unless extrauterine disease is clinically suspected. However, in one study, 34% of women with low-grade disease had preoperative imaging with CT or MRI despite these guidelines; these patients were discovered to have higher rates of false-positive results than true detection of extrauterine disease [17]. Similarly, in a study with men with low and intermediate-grade prostate cancer who had inappropriate staging imaging with CT or MRI, there was a 38% false-positive rate and a 10% incidental finding rate, with only 1% of patients demonstrating metastatic disease [18]. In this current study, we evaluated the use of pre-treatment CT and MRI scans in the workup of PDAC. We did not find an association between receiving an MRI and changing treatment plan (i.e., upfront surgery versus upfront chemotherapy). We did find an association between increased number of pre-treatment abdominopelvic CT scans and patients who underwent upfront surgical resection.

Limitations of this study include its use of retrospective claims-based data, which does not provide individual patient-level data that may have prompted a preoperative MRI or additional abdominopelvic CT scans in the surveillance period. For instance, initial inconclusive CT findings, small liver lesions, new symptoms, or rising CA 19-9 levels could all be reasons to warrant further scrutiny with supplemental imaging. Additionally, we could not differentiate between surveillance imaging and diagnostic imaging for symptoms in the surveillance analysis. We identified all imaging that was performed following surgery and identified the interval of time from surgery and the first imaging test, and subsequently thereafter between each imaging test. Our data lack staging information beyond T-stage (e.g., no data on nodal stage or metastasis). However, we assumed that if patients underwent a pancreatic surgery, they did not have metastasis. We cannot assess imaging quality or protocol adherence. There is also selection bias in who received an MRI and who did not, and subsequent unmeasured confounding. The military population captured in this data may not generalize to the civilian setting. Lastly, sample size might be underpowered for subgroup analyses.

## Conclusions

Imaging among patients with PDAC, both preoperatively and postoperatively in the surveillance phase after resection, remains an area of debate. Among patients who underwent multiple pre-treatment abdominopelvic CT scans, there was a significantly higher proportion of patients undergoing upfront surgical resection. More frequent surveillance imaging was associated with a hazard of death, and this could be explained by the use of more frequent imaging due to symptoms from early recurrence/metastasis. Additional research is needed to understand the impact of pre-treatment imaging and surveillance imaging on treatment plans and OS, respectively.

## Appendices

**Table 6:** Codes for diagnoses, surgery, chemotherapy, and imaging. ICD-10-CM = International Classification of Diseases, 10th Revision, Clinical Modification; CPT = Current Procedural Terminology

Diagnosis, surgery, chemotherapy, and imaging codes	
ICD-10-CM diagnosis codes	C250, C251, C252, C253, C257, C258, C259, D136, D137, D135, D3A8, C240, C241, C249, C170, C7A1, C7A8, C7889, C7A098, C221, K862
ICD-10 PCS (surgery) codes	0FBG0ZZ, 0FBG3ZZ, 0FBG4ZZ, 0FBG8ZZ, 0FTG0ZZ, 0FTG4ZZ, 0FCG0ZZ, 0FCG3ZZ, 0FCG8ZZ, 0FCG4ZZ, 0FBD0ZZ, 0FBD3ZZ, 0FBD4ZZ, 0FBD7ZZ, 0FBD8ZZ, 0FBF0ZZ, 0FBF3ZZ, 0FBF4ZZ, 0FBF7ZZ, 0FBF8ZZ, 0FCD0ZZ, 0FCD3ZZ, 0FCD4ZZ, 0FCD7ZZ, 0FCD8ZZ, 0FCF0ZZ, 0FCF3ZZ, 0FCF4ZZ, 0FCF7ZZ, 0FCF8ZZ, 0FTD0ZZ, 0FTD4ZZ, 0FTD7ZZ, 0FTD8ZZ, 0FTF0ZZ, 0FTF4ZZ, 0FTF7ZZ, 0FTF8ZZ
ICD-10-CM diagnosis codes for chemotherapy	Z79.63, Z79630, Z790631, Z790632, Z790633, Z79.634, Z79.64, Z79.69, Z79.8, Z79.899, Z5111
CPT codes for imaging modalities	
CT of the abdomen/pelvis	74150, 74160, 74170, 74176, 74177, 74178
MRI of the abdomen	74181, 74182, 74183

**Table 7:** Consort table

Consort table	
Pre-treatment imaging analysis	
Initial starting cohort of patient encounters	N = 3,860
Excluding patients who did not have surgery	N = 2,737 (removed 1123)
Excluding encounter duplications	N = 1,594 (removed 1143)
Excluding patients who did not have pre-treatment radiology imaging	N = 1,171 (removed 423)
Among the patients who received chemotherapy and surgery	N = 238 received chemotherapy as initial treatment; N = 933 received surgery as initial treatment
Among 933 who received surgery as initial treatment, excluding patients who did not have pre-treatment imaging. Among 238 who received chemotherapy as initial treatment, excluding patients who did not have pre-treatment imaging	N = 258 (removed 675); N = 44 (removed 194)
Total patient encounters	N = 302
Going from patient encounters to patients	N = 138 patients
Post-treatment surveillance analysis	
Initial starting cohort of patient encounters	N = 3,860
Excluding patients who did not have surgery	N = 2,737 (removed 1,123)
Excluding encounter duplications	N = 1,594 (removed 1,143)
Excluding patients who did not have pre-treatment radiology imaging	N = 1,171 (removed 423)
Excluding patients who did not have post-surgery imaging	N = 527 (removed 644)
Going from patient encounters to patients	N = 125 patients
